# Comprehensive Thermal Analysis of Diamond in a High-Power Raman Cavity Based on FVM-FEM Coupled Method

**DOI:** 10.3390/nano11061572

**Published:** 2021-06-15

**Authors:** Zhenxu Bai, Zhanpeng Zhang, Kun Wang, Jia Gao, Zhendong Zhang, Xuezong Yang, Yulei Wang, Zhiwei Lu, Richard P. Mildren

**Affiliations:** 1 Center for Advanced Laser Technology, Hebei University of Technology, Tianjin 300401, China; zxbai@hebut.edu.cn (Z.B.); gaojia20201@163.com (J.G.); wyl@hebut.edu.cn (Y.W.); zhiweilv@hebut.edu.cn (Z.L.); 2 Hebei Key Laboratory of Advanced Laser Technology and Equipment, Tianjin 300401, China; 3 MQ Photonics Research Centre, Department of Physics and Astronomy, Macquarie University, Macquarie Park, NSW 2109, Australia; rich.mildren@mq.edu.au; 4 School of Energy and Environmental Engineering, Hebei University of Technology, Tianjin 300401, China; 181063@stu.hebut.edu.cn (Z.Z.); 201821301010@stu.hebut.edu.cn (Z.Z.); 5 Hangzhou Institute for Advanced Study, UCAS, Hangzhou 330106, China; xuezong.yang@ucas.ac.cn

**Keywords:** diamond, thermal analysis, high-power, Raman laser, FVM-FEM

## Abstract

Despite their extremely high thermal conductivity and low thermal expansion coefficients, thermal effects in diamond are still observed in high-power diamond Raman lasers, which proposes a challenge to their power scaling. Here, the dynamics of temperature gradient and stress distribution in the diamond are numerically simulated under different pump conditions. With a pump radius of 100 μm and an absorption power of up to 200 W (corresponding to the output power in kilowatt level), the establishment period of thermal steady-state in a millimeter diamond is only 50 μs, with the overall thermal-induced deformation of the diamond being less than 2.5 μm. The relationship between the deformation of diamond and the stability of the Raman cavity is also studied. These results provide a method to better optimize the diamond Raman laser performance at output powers up to kilowatt-level.

## 1. Introduction

High-power lasers with high beam quality are of interest for applications such as material processing, space exploration, defence, and high-energy physics [1,2,3]. However, the negative effects, including thermal lens, birefringence, beam distortion, and spectral broadening caused by the intensified thermal accumulation in the laser media during power scaling, limit improvements to its performance [4,5,6]. At present, optimization of pump efficiency [7,8], increases in the surface area of the gain media (viz. fiber laser, slab laser, disk laser, etc.) [9,10,11], improvements in the heat dissipation capacity (viz. liquid nitrogen cooling, microchannel cooling, etc.) [12,13], and beam combination [14,15,16,17], are the main approaches used to alleviate or avoid thermal effects in high-power lasers. However, the mitigation of thermal effects through the above indirect approaches is still restricted by the inherent optical and thermophysical properties of the working material. Therefore, it is effective to improve the output limit of high-power lasers using optical materials with better photothermal properties.

In the last decade, diamond has been of interest to the laser community due to its excellent thermophysical, spectral, and nonlinear optic properties. By using a single crystal diamond with a volume of less than 0.04 cm^3^, over a kilowatt of quasi-continuous Raman laser power (in thermal quasi-steady-state) [18,19] and high power (11 W for continuous-wave, and on-time power 40 W for quasi-continuous-wave) Brillouin lasing [20,21] have been demonstrated. In addition, lasers based on diamond as the gain medium provide a promising approach to high brightness beam generation [22,23] with a wide spectral range [24,25,26]. Recently, thermal-induced lensing was observed in diamond in a high-power device [18,19], despite the thermal conductivity being higher than most other laser media by over a factor of 100. Numerical simulation of thermal effects is vital for understanding the behavior of high-power diamond lasers and providing guidance for cavity optimization and cooling as laser powers increase.

Regarding the numerical simulation, the finite volume method (FVM) and the finite element method (FEM) are two widely used approaches to represent and solve partial differential equations in the form of algebraic equations. Both FVM and FEM are implemented based on the space discretization with numerous meshes and nodes, and the temporal discretization with a series of time steps (for unsteady problems). In FVM, “finite volume” refers to the small volume surrounding each node on a mesh, and all of the divergence terms in the partial differential equations are evaluated as fluxes in the surfaces of each finite volume by the volume integrals in the partial differential equations, with divergence terms being converted to surface integrals. The exact expression of the average value of the solution in each finite volume can be evaluated by FVM, based on which, the solution to the whole volume can be approximated [27,28]. In FEM, a large system is divided into a great deal of smaller parts, which are referred to as “finite elements”, and local approximations of a solution are made using the local data for each finite element, which are stitched together to create the global approximation for the whole system [29]. In general, FVM is more suitable for the flow and heat transfer problem, while FEM is better at stress–strain analysis. In some previous studies, the two approaches of FVM and FEM are always combined to solve the thermal–fluid–mechanical coupled problems, such as those in solar receivers [30,31].

In this paper, the coupled FVM and FEM are used to simulate the evolution of temperature distribution and thermal deformation in diamond in a high-power operation Raman cavity. Meanwhile, the relationship between the deformation of diamond and temperature gradient is studied. Finally, the influence of the change in intracavity beam radius induced by thermal deformation on the threshold of the Raman oscillator is analyzed. As far as we know, this is the first time the thermal effect of a diamond is quantitively analyzed by numerical study.

## 2. Physical Model and FVM-FEM Coupled Numerical Method

### 2.1. Diamond Raman Laser (DRL) Setup for the Simulation

**Figure 1:**
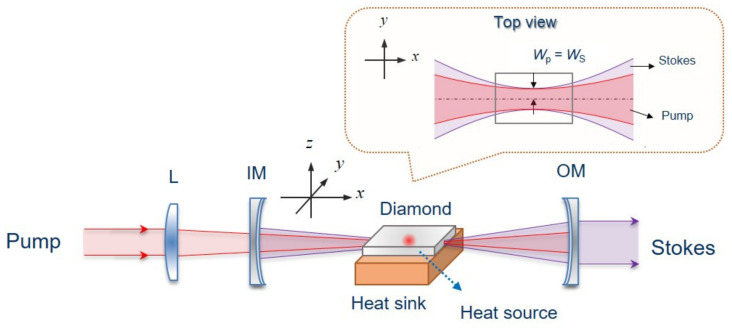
Illustration of the DRL for simulation (inset: top view of the beam propagation in the diamond).

The numerical study was performed for the DRL reported in References [22,23,32,33], where an 8 mm × 4 mm × 1.2 mm single-crystal diamond was used. A schematic of the external-cavity DRL, including the propagation diagram of pump light and Stokes light, is shown in Figure 1. The DRL was pumped by a 1064 nm linearly polarized laser that focused on the diamond using a focus lens. The diamond was placed in a near-concentric cavity, where the pump beam radius *w_p_* equals the cold-cavity fundamental Stokes mode waist radius *w_s_* for good mode-matching. The concave input-coupling mirror (IM) has high transmission at the pump wavelength (1064 nm) and high reflectivity at the first Stokes wavelength (1240 nm); the concave output-coupling mirror (OM) is highly reflective at 1064 nm and has partial transmission at 1240 nm. The radius of curvature of both mirrors is 100 mm. The ends of the diamond are anti-reflection-coated at 1240 nm. A copper heat sink is placed under the diamond; meanwhile, cooling water is supplied to remove the heat in the heat sink, and the amount of cooling water is assumed to be sufficient to keep the bottom surface of heat sink at a constant temperature of 298 K.

The parameters of the diamond and setup used in the simulation are listed in Table 1. The thermal energy generated by the Raman cavity is assumed to be a spherical heat source with a radius of 40–100 μm (approximating the cold-cavity fundamental mode radius at the Stokes) located in the center of the diamond. The heat introduced to the diamond was mainly caused by the absorption and quantum defect (*η* = *λ*_p_/*λ*_s_) in the process of Raman conversion. The total power absorbed was set at 200 W at the maximum, corresponding to the output power over 1000 W (deduced from References [19,33]). The heat conduction in the diamond and the copper heat sink, the air natural convection, and the thermal radiation between the heated diamond/copper and the surroundings are all taken into account. Both steady-state simulations and transient simulations regarding the above heat transfer processes were performed by ANSYS Fluent based on FVM. Furthermore, the thermal deformation of the diamond induced by the large temperature gradient was simulated by the ANSYS Static Structural based on FEM.

### 2.2. Thermal Model

For the solid domain (including the diamond and the copper sink), the governing equation, i.e., the energy equation, is expressed as follows,

(1) $$\frac{\partial T}{\partial \tau }=\frac{\lambda_{s}}{\rho_{s}c}\frac{\partial T^{2}}{\partial x_{i}^{2}}+\frac{S_{h}}{\rho_{s}c_{p,s}}$$

where *ρ*_s_, *λ*_s_, and *c_p_*_,s_ are the density, thermal conductivity, and specific heat capacity of the diamond or the copper sink, respectively; *T* denotes temperature, *τ* represents time, and *x_i_* (*i* = 1–3) represents the three coordinates in the Cartesian coordinate system; *S*_h_ is the heat source generated by the dissipation of the laser, which occurs in a sphere with a certain diameter located in the center of the diamond.

For the fluid domain i.e., the air around the diamond and the sink, the governing equations include the continuity equation, the momentum equation, and the energy equation, which were expressed as follows

(2) $$\frac{\partial \rho_{a}}{\partial \tau }+\frac{\partial }{\partial x_{i}}(\rho_{a}v_{i})=0$$

(3) $$\frac{\partial \left(\rho_{a}v_{j}\right)}{\partial \tau }+\frac{\partial }{\partial x_{i}}(\rho_{a}v_{i}v_{j})=-\frac{\partial p}{\partial x_{i}}+\frac{\partial }{\partial x_{j}}\left[\mu_{a}\left(\frac{\partial v_{i}}{\partial x_{j}}+\frac{\partial v_{j}}{\partial x_{i}}\right)\right]+\rho_{a}g_{i}$$

(4) $$\frac{\partial \left(\rho_{a}T\right)}{\partial \tau }+\frac{\partial }{\partial x_{i}}(\rho_{a}v_{i}T)=\frac{\lambda_{a}}{c_{p,a}}\frac{\partial }{\partial x_{i}}\left(\frac{\partial T}{\partial x_{i}}\right)$$

where *ρ*_a_ is the density of air that is assumed to be incompressible ideal gas, and *c_p,_*_a_ is the specific heat capacity of air; *g_i_* is the acceleration of gravity, and *p* represents pressure.

The boundary conditions were set as follows. The bottom surface of the copper sink is set at a constant wall temperature of 298 K, with the assumption that the cooling capacity of the cooling water is infinite. The interfaces between the air domain and the solid domain were set as coupled surfaces. The boundaries of the air domain were specified as pressure outlet conditions.

In the present study, the transient characteristics during the heating process and cooling process are investigated. For the heating process, the initial condition was set as the air being stationary and the temperatures of the diamond and the copper sink being the same as the surrounding air. The steady simulation results from the heating process were regarded as the initial conditions for the cooling process.

The governing equations for the thermal model were discretized by FVM and solved by the software ANSYS Fluent. The mesh in the diamond is specially refined. According to the grid-independent test and the time-step independent study, the grid system with 28,667,369 elements and the time step of Δ*τ* = 1 μs were selected. In addition, the radiation heat transfer in the computational domain was simulated by the discrete ordinate (Do) model. 

### 2.3. Thermo-Elasticity Model

The diamond and the copper sink are regarded as thermo-elasticity with isotropic physical properties. The basic equations of thermo-elasticity, including the equilibrium differential equations, the strain-displacement equations, and the constitutive equations, are expressed as follows.

Equilibrium differential equations:

(5) $$\left\{\begin{array}{l} \frac{\partial \sigma_{x}}{\partial x}+\frac{\partial \tau_{yx}}{\partial y}+\frac{\partial \tau_{zx}}{\partial z}=0\\ \frac{\partial \sigma_{y}}{\partial y}+\frac{\partial \tau_{zy}}{\partial z}+\frac{\partial \tau_{xy}}{\partial x}=0\\ \frac{\partial \sigma_{z}}{\partial z}+\frac{\partial \tau_{xz}}{\partial x}+\frac{\partial \tau_{yz}}{\partial y}=0 \end{array}\right.$$

According to the equivalence theorem of shear stress, the following relations are obtained:

(6) $$\tau_{yz}=\tau_{zy},\;\tau_{zx}=\tau_{xz},\;\tau_{xy}=\tau_{yx}$$

Strain-displacement equations:

(7) $$\left\{\begin{array}{l} \varepsilon_{x}=\frac{\partial u_{x}}{\partial x},\;\varepsilon_{y}=\frac{\partial u_{y}}{\partial y},\;\varepsilon_{z}=\frac{\partial u_{w}}{\partial z}\\ \gamma_{xy}=\frac{\partial u_{x}}{\partial y}+\frac{\partial u_{y}}{\partial x}\\ \gamma_{yz}=\frac{\partial u_{y}}{\partial z}+\frac{\partial u_{z}}{\partial y}\\ \gamma_{zx}=\frac{\partial u_{z}}{\partial x}+\frac{\partial u_{x}}{\partial z} \end{array}\right.$$

Constitutive equations for thermal elasticity

(8) $$\left\{\begin{array}{l} \varepsilon_{x}=\frac{1}{E}\left[\sigma_{x}-\mu \left(\sigma_{y}+\sigma_{z}\right)\right]+\alpha \Delta T\\ \varepsilon_{y}=\frac{1}{E}\left[\sigma_{y}-\mu \left(\sigma_{z}+\sigma_{x}\right)\right]+\alpha \Delta T\\ \varepsilon_{z}=\frac{1}{E}\left[\sigma_{z}-\mu \left(\sigma_{x}+\sigma_{y}\right)\right]+\alpha \Delta T\\ \gamma_{xy}=\frac{\tau_{xy}}{G},\;\gamma_{yz}=\frac{\tau_{yz}}{G},\;\gamma_{zx}=\frac{\tau_{zx}}{G} \end{array}\right.$$

where *E* is the Young’s modulus, *μ* is the Poisson’s ratio, *α* is the thermal expansion coefficient, ∆*T* is the temperature rise provided by the thermal modeling, and *G* represents the shear modulus and is expressed as

(9) $$G=\frac{E}{2\left(1+\mu \right)}$$

Both the diamond and the copper sink were allowed to freely expand, except the interface between the diamond and sink, which was set as bonded.

The governing equations for the thermo-elasticity model were solved by the software ANSYS Static Structural, based on FEM. According to the grid-independent test, the grid system with 113,187 elements was adopted.

## 3. Results and Discussion

### 3.1. Transient Response and Temperature Distribution

**Figure 2:**
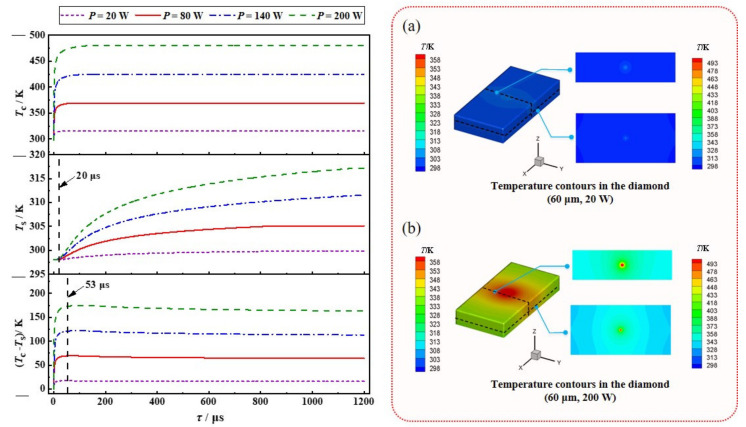
Temperature variation of diamond under different thermal powers in the heating process (top: center, middle: upper surface, and bottom: the difference). Inset: the temperature contours in the diamond with the heat source radius of 60 μm and power of (**a**) 20 W, and (**b**) 200 W, respectively.

Since the temperature gradient of diamond crystal is an important factor leading to its deformation and thermal lens effect, the temperature response characteristics of diamond in the heating stage was studied first. The influence of the heat source power on the transient temperature of the diamond was numerically studied, with a heat source radius of 60 μm. Figure 2 shows the evolution of the temperature of the diamond over time when suffering from various thermal powers, where the temperatures in the volume center (*T*_c_) and on the center point of the upper surface (*T*_s_) are monitoredand taken as an example. The temperature gradient represented by the temperature difference between the volume center and the upper surface, *T*_c_–*T*_s_, is correspondingly illustrated in Figure 2. The inset (a) and (b) of Figure 2 further represent the temperature distribution of diamond crystal under steady-state at the heat sources of 20 W and 200 W, respectively.

Once the heat source is loaded, the temperature in the volume center (*T*_c_) where the heat source is located increased immediately. The surface temperature (*T*_s_) remained at the initial temperature of 298 K, until the heat located in the center was transferred to the surface after about 12 μs. It is noted that it took a relatively long time (greater than 0.5 s, not illustrated in Figure 2) to approach 99% of the steady-state values for both center temperature (*T*_c_) and the surface temperature (*T*_s_). However, the temperature gradient reached 99% of the steady-state value after a relatively short time (53 μs). As the thermal effect in the external-cavity diamond Roman laser is mainly driven by a temperature gradient rather than the local temperature in the diamond crystals, the evolution of the temperature gradient is of more significance than the local temperature [34] and, thus, the thermal constant time is defined as the time taken for the temperature gradient to reach 99% of the steady-state value. Therefore, it can be observed from Figure 2 that the thermal constant time is 53 μs for a constant heat source, with a radius of 60 μm, and the thermal constant time was independent of the heat source through all the power ranges, which is consistent with the previous report in Reference [32]. This means that it is sufficient to enable the establishment of steady-state temperature gradients under various heat sources after a period of on-time durations of 53 μs, which can be representative of continuous-wave operation. Although the local temperature continues to rise, the local temperature rise within several hundred Kelvin is not expected to significantly affect the Raman conversion due to the weak effects on the Raman line-shape and gain coefficient [34]. In addition, although the thermal constant time is independent of the heat sources, the steady-state value of the temperature gradient in the diamond crystal is proportional to the heat sources, and the thermal deformation induced by temperature gradient consequently increases with the heat sources, which will be discussed in Section 3.2.

**Figure 3:**
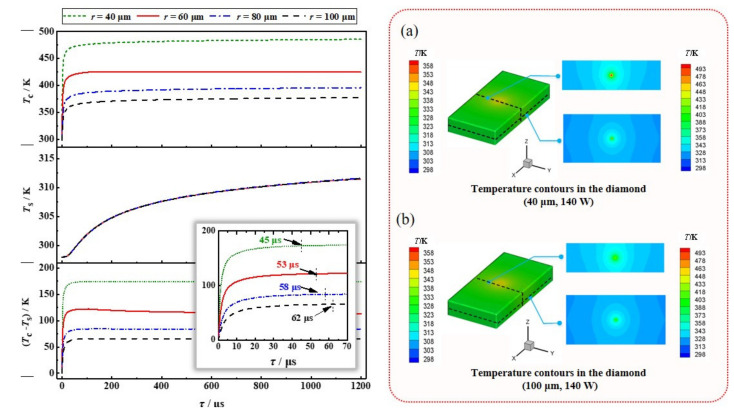
Temperature variation of diamond with different thermal radius in the heating process (top: center, middle: upper surface, and bottom: the difference). Inset: the temperature contours in the diamond with the absorbed power of 140 W and heat source radius of (**a**) 40 μm, and (**b**) 100 μm, respectively.

Figure 3 shows the influence of the beam waist radius on the transient temperatures of diamond when the absorbed power is constant (140 W, corresponding to the theoretical output power over 1000 W according to Reference [35]). Inset (a) and (b) represent the temperature distribution of diamond crystal with the heat source radius of 40 μm and 100 μm, respectively. The temperature in the center of the diamond is inversely proportional to the waist radius of the heat source; however, the temperature of diamond surface is not affected by the size of heat source. Furthermore, Figure 3 indicates that the thermal constant time depends on the waist radius of the heat source. When the radii of the heat source are 40, 60, 80 and 100 μm, the thermal constant times are 45, 53, 58 and 62 μs, respectively. This finding proves that it takes slightly longer for the thermal effects in the DRL with a smaller waist radius pump beam to become established and stable.

**Figure 4:**
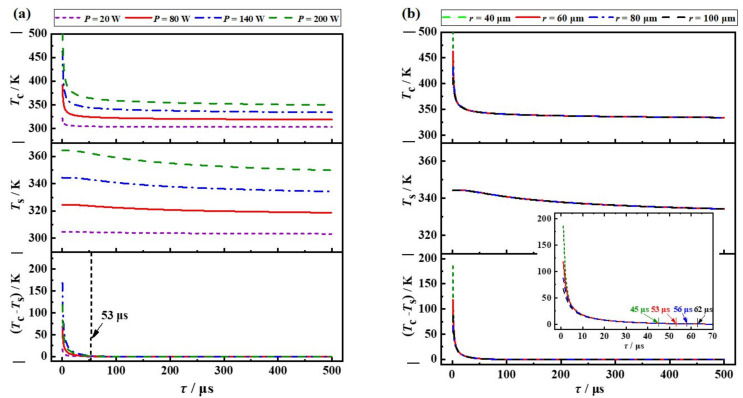
Temperature variation in diamond in the cooling process with (**a**) different absorbed power (60 μm thermal radius), and (**b**) different thermal radius (140 W power), respectively.

To understand the maximum repetition rate of DRL under quasi-continuous pumping conditions, the temperature variation in a diamond is calculated after the heat source is removed, when the thermal steady-state is realized. Figure 4a,b shows the cooling process of the diamond after thermal state-stability is reached, when the radii of the heat source and the absorbed power are constant (refer to Figure 2 and Figure 3), respectively, where the steady-state temperature distributions in the diamond crystal under various pump beams with different waist radii and different heat sources powers are regarded as the corresponding initial state. For the reason discussed above, we still pay more attention to the evolution of the temperature gradient in the diamond crystal. Figure 4a indicates that temperature gradient drops to 1% of the values of the initial state (approaching 0 K) after the laser stops working for 53 μs for the cases with different heat source powers, corresponding to their thermal constant times. This means that the temperature-gradient-driven thermal effects in the external-cavity DRL under a pump beam with a waist radius of 60 μm almost disappear after 53 μs. Similarly, for the cooling process with different waist radii, as illustrated in Figure 4b, it takes the same time as their respective “thermal constant time” for the temperature gradients to drop to 1% of their respective initial state values in different cases.

**Figure 5:**
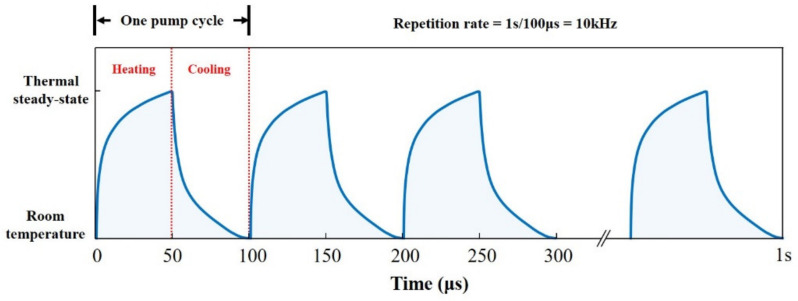
The diagram of temperature change in diamond at repetitive operation without heat accumulation.

In summary, thanks to the extremely high thermal conductivity of the diamond, the thermal constant time in the DRL is quite short, ranging from 45 to 60 μs in typical application conditions. The waist radius is the most important factor to determine the thermal constant time of diamond, and the pump beam power has no effect on this. As illustrated in Figure 5, the diamond reaches the thermal steady-state after 50 μs pumping (heating period); once the pump stops, the temperature of diamond drops to room temperature after 50 μs (cooling period). It can be concluded that, for a single-sided cooled diamond crystal as the gain medium, long-term kW-level Raman lasing without heat accumulation can be realized at a repetition rate of up to 10 kHz.

### 3.2. Thermal Deformation Model

**Figure 6:**
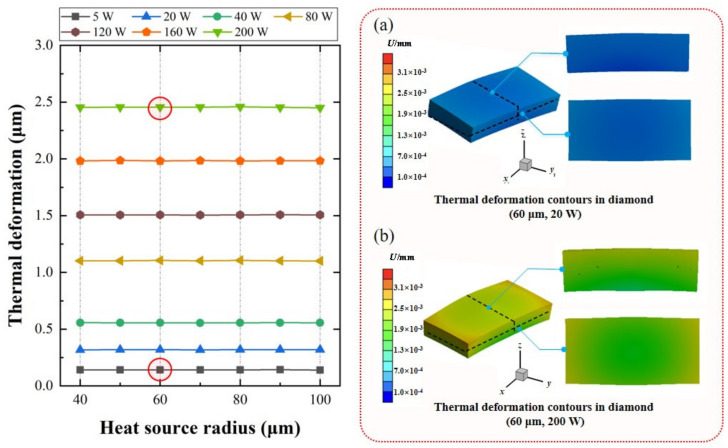
Thermal deformation contours in diamond with different power and heat source radius. Inset: (**a**) 20 W, and (**b**) 200 W with hear source radius of 60 μm, respectively.

The deformation of the optical crystal caused by the thermal effect is the main reason for the thermal lens effect, which leads to fracture damage [36,37]. Figure 6 illustrates the thermal deformation contours in diamond with different steady-state power and heat source radii. The results show that the thermal deformation is only related to the absorbed power, not to the heat source radius. The inset (a) and (b) show the thermal deformation of diamond, with absorbed powers of 20 W and 200 W, respectively. With the maximum absorbed power of 200 W, the maximum deformation of diamond is only 2.456 ± 0.004 μm with the heat source radius changing from 40 to 100 μm. Based on the ray-tracing method, a 2.5 μm deformation of the diamond results in the thermal lensing *f* = 54.5 mm. While the DRL works at the hundred-watt level (0.319 ± 0.004 μm deformation with absorbed power 20 W), the thermal effect is nearly negligible, with *f* = 413.9 mm at maximum.

**Figure 7:**
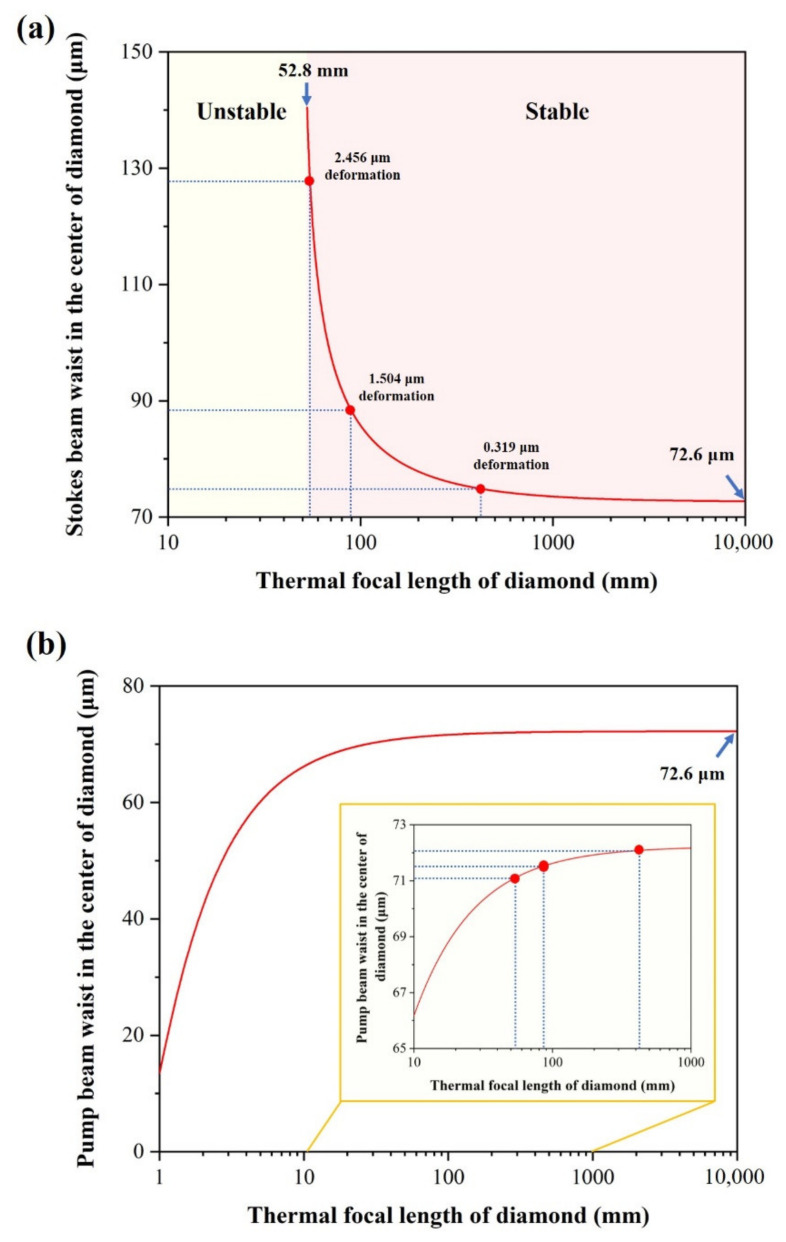
(**a**) Stokes beam waist, and (**b**) pump beam waist as a function of the thermal focal length of diamond.

To further study the influence of the thermal effect on the cavity mode, we calculated the influence of the thermal lens effect’s strength on the intracavity Stokes mode. According to the schematic diagram in Figure 1, the cold cavity length is set as 201 mm, which corresponds to the Stokes beam waist radius of 72.6 μm. As can be seen from Figure 7a, the increased thermal-induced deformation will cause a stronger thermal lensing effect (corresponding to shorter *f*), which leads to an increase in the Stokes beam radius in the center of the diamond, and then to an increased lasing threshold. When the thermal lens effect is intensified further (*f* < 52.8 mm), the resonator will become unstable. In contrast, the radius of pump beam in the center of the diamond is slightly inversely proportional to the intensity of the thermal effect. However, neither of the above conditions poses any significant challenge to the generation of highly efficient diamond Raman lasing. For example, when *f* = 54.5 (2.5 μm deformation), we need to increase the cavity length from 201 mm to ~204.4 mm to keep the radius of the pump and Stokes constant (~72.6 μm). Moreover, the waist radius of the Stokes beam is usually set to be no smaller than that of the pump beam for high-quality Raman lasing; therefore, the simulation results also indicate that the output Stokes can still maintain high quality, even in the presence of thermal effects.

## 4. Conclusions and Outlook

In this paper, based on the FVM-FEM coupled numerical method, we study the temperature evolution of diamond crystal with time under high power operation, and the influence of shape variables on the mode of the resonator. The results show that the time for the diamond to reach the thermal steady-state is independent of the absorbed power, and only positively related to the waist radius of the Stokes beam in the cavity. When the output power of the DRL is in the order of kW, the change of the beam waist of the resonator caused by the small deformation of the diamond can be easily compensated by tuning the length of the resonator. Our study builds a bridge between the design and the operation power of the diamond Raman laser, and is of great significance to realize the high-efficiency output at powers in the thermal-affected regime. Meanwhile, this work lays a theoretical foundation for the operation and thermal management of high-power DRLs.

## Figures and Tables

**Table 1:** Parameters of the diamond and setup for simulation.

	Parameters	Value
Diamond	Thermal conductivity	2200 W/(m·K)
	Coefficient of thermal expansion	10^−6^ K^−1^
	Spectral transmission range	>2 μm
	Density	3.51 g/cm^3^
	Specific heat capacity	0.519 J/(g·K)
	Refractive index	2.35 @1 μm
	Size and volume	8 mm × 4 mm × 1.2 mm & 0.0384 cm^3^
Copper	Thermal conductivity	385 W/(m·K)
	Temperature	298 K (25 °C)
Variable parameter of the DRL	Pump beam radius	40, 60, 80, 100 μm
	Stokes beam radius	40, 60, 80, 100 μm
	Absorbed power	5, 20, 40, 80, 120, 160, 200 W
